# Association between polymorphisms in interleukin-18 promoter and risk of coronary artery disease: a meta-analysis

**DOI:** 10.1042/BSR20192721

**Published:** 2019-11-19

**Authors:** Zheng Lian, Su-Fang Li, Yu-Xia Cui, Man-Yan Wu, Li-Na Su, Dan Hu, Wei-Jue Xiong, Hong Chen

**Affiliations:** 1 Department of Cardiology, Peking University People’s Hospital, Beijing, China; 2 Beijing Key Laboratory of Early Prediction and Intervention of Acute Myocardial Infarction, Peking University People’s Hospital, Beijing, China; 3 Center for Cardiovascular Translational Research, Peking University People’s Hospital, Beijing, China

**Keywords:** coronary artery disease, interleukin-18, meta-analysis, polymorphisms

## Abstract

**Background:** Previous studies have explored associations between interleukin-18 (IL-18) promoter polymorphisms and coronary artery disease (CAD). However, the results were controversial. We conducted a meta-analysis to clarify the association between the two polymorphisms and CAD risk. **Methods:** We searched English and Chinese databases and calculated the odds ratio (OR) and 95% confidence interval (CI) to estimate whether there are genetic associations between IL-18 promoter polymorphisms and the risk of CAD. All relevant studies were screened and meta-analyzed using STATA 15.0. **Results:** A total of 15 studies, including 12 studies for -137 G/C and 9 studies for -607 C/A, were identified for the meta-analysis. For -137 G/C, the results showed a significantly reduced risk of CAD in the dominant model (OR = 0.85) and heterozygous model (OR = 0.88) in the overall analysis. However, in subgroup analysis, decreased CAD risks were only observed in Asian populations for heterozygous genetic models. For -607 C/A, the overall OR revealed a reduced risk of CAD in all five genetic models (allelic, OR = 0.78; recessive, OR = 0.75; dominant, OR = 0.68; homozygous, OR = 0.61; heterozygous, OR = 0.72). In subgroup analysis, reduced CAD risk was also found in five genetic models of the Asian population. We also found that the IL-18 polymorphisms were correlated with myocardial infarction (MI) and multivessel (MV) disease. **Conclusion:** Our results suggested that the -137 polymorphism and -607 polymorphism in the IL-18 promoter were negatively associated with CAD, especially in the Asian population. In addition, some genetic models were correlated with the severity of CAD.

## Introduction

Coronary artery disease (CAD) is an important cause of cardiovascular mortality worldwide [1,2]. CAD includes a group of diseases such as angina, sudden death and myocardial infarction (MI). Atherosclerotic plaques have the main role in the progression of CAD, which are also associated with both innate and adaptive immune responses [3]. Growing evidence indicates that increased levels of circulating pro-inflammatory cytokines could further amplify the CAD risk [4].

The inflammatory response could promote the formation and stability of plaques [5]. Previous studies have indicated that several inflammatory factors contribute to the development of CAD, such as C-reactive protein (CRP) and tumor necrosis factor α (TNF-α) [6,7]. In addition, several new cytokines have been identified to be associated with the development of CAD, such as interleukin-18 (IL-18) [4]. IL-18 was originally identified as an IFN-γ-inducing factor (IGIF). IL-18 mRNA is expressed in a wide range of cells, including Kupffer cells, macrophages, T cells, B cells, osteoblasts, keratinocytes, dendritic cells, astrocytes and microglia [8,9]. Previous studies have shown that the level of plasma IL-18 was significantly elevated in CAD patients [10–12] and that the level of IL-18 could be a biomarker to predict the prognosis of CAD [13].

The gene for human IL-18 is located on chromosome 11q22.2–22.3 and contains six exons. Within the promoter region of the *IL-18* gene, substitution of G>C at position 137 changes a histone 4 transcription factor-1 (H4TF-1) nuclear factor-binding site, while a change of C>A at position 607 disrupts a cyclic adenosine monophosphate (cAMP) responsive element protein-binding site. These changes influence the transcriptional activity of the *IL-18* gene [14]. Indeed, numerous case–control studies [15–28] have investigated whether polymorphisms at position -137 (rs187238) or -607 (rs1946518) within the IL-18 promoter influence the risk of CAD, but the results were inconclusive and contradictory, prompting us to perform a comprehensive meta-analysis of all available evidence on these potential associations.

## Materials and methods

### Literature search strategy

PubMed, EMBASE, Google Scholar, Cochrane Central Register of Controlled Trials (CENTRAL), the Chinese National Knowledge Infrastructure (CNKI) and Chinese Biomedical Literature Database (CBM), databases were systematically searched for clinical and experimental case–control studies of association between CAD and the -137 polymorphism (rs187238) and/or the -607 polymorphism (rs1946518) in the IL-18 promoter and that were published in English or Chinese up to 10 July 2019. The following search strings were used: interleukin-18 -137; interleukin-18 -607; IL-18 -137; IL-18 -607; rs187238; rs1946518; these six terms in combination with polymorphism, polymorphisms, SNP, variant, variants, variation, genotype, genetic or mutation; and all of the above terms in combination with CAD or CHD or coronary heart disease or coronary artery disease or myocardial infarction or angina or sudden death. Reference lists in identified articles and reviews were also searched manually to identify additional eligible studies.

### Inclusion and exclusion criteria

If the obtained studies fulfilled the following criteria, they were identified as eligible: (1) case–control design; (2) research on the association between polymorphisms in the IL-18 promoter and risk of CAD; (3) sufficient published genotype frequencies data to estimate the odds ratio (OR) and 95% confidence interval (CI). The exclusion criteria included the following: (1) the genotype frequency data were unavailable; (2) animal model research; (3) review articles, case reports, meta-analysis; (4) overlapping publications (the studies with more subjects or recently published were included).

### Quality assessment

The quality scoring criteria were modified from previous literature, and the score ranged from 0 to 9 points (Table 1) [29]. Two independent investigators (Su and Song) evaluated the quality of articles according to the modified criteria. A study with a score of ≥6 was defined as high quality, while one with a score <6 was low quality.

**Table 1:** The criteria for quality assessment

Criteria	Score
**Representativeness of Cases**	
Continuous collection and representative cases within clearly defined limits	2
With potential selection bias	1
Not described	0
**Source of Controls**	
Population-based	2
Hospital-based	1
Not described	0
**Hardy–Weinberg Equilibrium in Controls**	
Hardy–Weinberg equilibrium	2
Hardy–Weinberg disequilibrium	1
**Genotyping Examination**	
Genotyping done under ‘blinded’ condition	1
Unblinded done or not mentioned	0
**Statistical Methods**	
Appropriate statistics and adjustment for confounders 2	2
Appropriate statistics but without adjustment for confounders	1
Inappropriate statistics used	0

### Statistical analysis

To assess the strength of the association between the two polymorphisms in the IL-18 promoter and CAD risks, the ORs with corresponding 95% CIs served as the effect size. For the -137 polymorphism (rs187238), the allelic (C vs. G), recessive (CC vs. GC+GG), dominant (GC+CC vs. GG), homozygous (CC vs. GG) and heterozygous (GC vs. GG) genetic models were used to obtain pooled ORs. For the -607 polymorphism (rs1946518), the allelic (C vs. A), recessive (AA vs. CA+CC), dominant (CA+AA vs. CC), homozygous (AA vs. CC) and heterozygous (CA vs. CC) genetic models were used to obtain pooled ORs. The subgroup analysis was performed according to the ethnicity and Hardy–Weinberg equilibrium (HWE) status of controls. Cochran’s Q statistic and *I^2^* test were used to assess the heterogeneity between different studies [30]. Heterogeneity was acceptable when the *P*-value was more than 0.10 and *I^2^* was <50%, and a fixed-effects model (the Mantel–Haenszel method) was used. In contrast, ORs were calculated by the random-effects model (DerSimonian and Laird method) [31,32]. Sensitivity analysis was performed to assess the effect of individual studies on pooled results and the stability of the results. The publication bias was detected using Begg’s funnel plot and Egger’s linear regression method [33]. All statistical analyses were performed using STATA 15.0 software with two-sided *P*-values. A *P*-value <0.05 was considered significant.

## Results

### Description of studies

**Figure 1:**
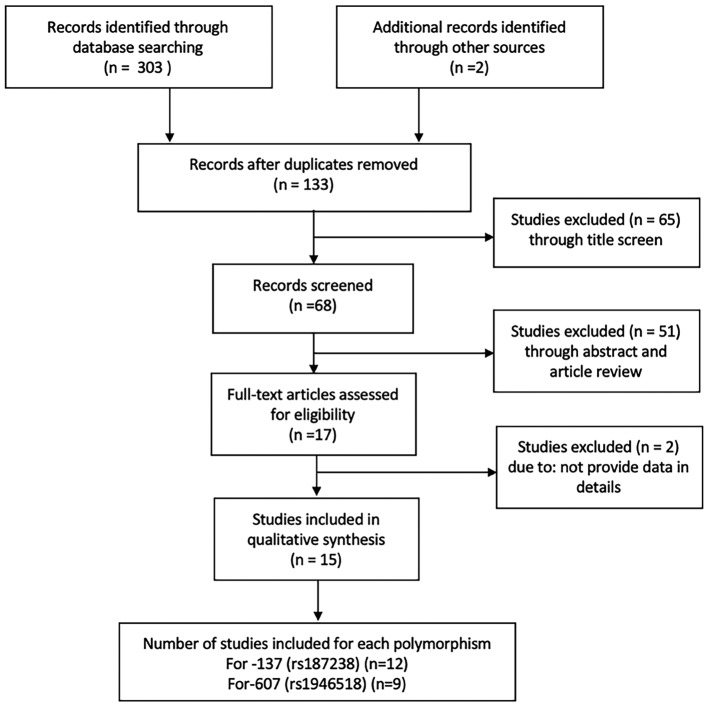
Flow chart of study selection

Three hundred and three articles were retrieved from the initial database search, 133 studies were screened for duplicates, case reports, *in vitro* or *in vivo* studies, meta-analysis, reviews, and the remaining articles (*n*=17) for secondary screening. Two articles were excluded in the secondary screening due to insufficient data. Finally, a total of 15 articles were identified, 12 of which considered the -137 polymorphism (rs187238) and 9 considered the -607 polymorphism (rs1946518) (Figure 1). The characteristics of the studies in the meta-analysis are shown in Table 2.

**Table 2:** Characteristics of studies in the meta-analysis

First author	Year	Ethnicity	Country	Genotyping method	Type of control	*P* for HWE	Cases/ Controls	Number of cases			Number of controls			Quality score
IL-18-137 (rs 187238)								GG	CG	CC	GG	CG	CC	
Bazgir, A. [15]	2018	Non-Asian	Iran	PCR-SSP	Healthy	0.183	314/364	168	122	24	160	171	33	7
Mitrokhin, V. [28]	2018	Non-Asian	Russia	PCR-Taqman	Patients without CAD	0.853	176/116	86	76	14	52	48	12	7
Fatemeh, H. [23]	2018	Non-Asian	Iran	PCR	Healthy	0.242	100/100	57	39	4	48	46	6	7
Jabir, N.R.	2017	Non-Asian	Saudi Arabia	PCR	Healthy	<0.01	76/74	48	19	9	49	16	9	6
Buraczynska, M. [24]	2016	Non-Asian	Poland	PCR-Taqman	Healthy	<0.01	1103/590	439	562	102	250	306	34	8
Kumar, R. [18]	2015	Asian	India	PCR	Healthy	0.533	300/300	168	102	30	176	105	19	7
Zhang, X. [27]	2011	Asian	China	PCR-SSP	Patients without CAD	0.439	468/432	352	112	4	308	116	8	6
Kariž, S. [19]	2011	Non-Asian	SLOVENIA	PCR- RFLP	Patients without CAD	0.301	169/326	90	71	8	162	141	23	8
Opstad, T.B. [22]	2011	Non-Asian	Norway	PCR-Taqman	Healthy	0.768	1001/204	532	394	69	108	82	14	7
Shayan, S. [25]	2009	Non-Asian	Iran	PCR	Patients without CAD	0.941	268/140	135	111	22	60	63	17	7
Pei, F. [20]	2009	Asian	China	PCR-SSP	Patients without CAD	0.203	234/216	180	53	1	150	63	3	8
Liu, W. [21]	2009	Asian	China	PCR	Patients without CAD	0.236	241/145	195	46	0	99	44	2	6

IL-18-607 (rs 1946518)								CC	CA	AA	CC	CA	AA	
Bazgir, A. [15]	2018	Non-Asian	Iran	PCR-SSP	Healthy	0.494	314/364	109	153	52	136	178	50	7
Jabir, N.R.	2017	Non-Asian	Saudi Arabia	PCR	Healthy	<0.01	74/74	65	0	9	59	2	13	6
Ma, J.B. [17]	2016	Asian	China	PCR- RFLP	Healthy	0.53	326/326	90	128	108	43	158	125	6
Kariž, S. [19]	2011	Non-Asian	SLOVENIA	PCR- RFLP	Patients without CAD	0.895	169/326	55	86	28	109	158	59	8
Zhang, X. [27]	2011	Asian	China	PCR-SSP	Patients without CAD	0.616	468/432	170	210	88	90	220	122	6
Opstad, T.B. [22]	2011	Non-Asian	Norway	PCR-Taqman	Healthy	0.762	1001/204	364	500	132	74	96	34	7
Shayan, S. [25]	2009	Non-Asian	Iran	PCR	Patients without CAD	0.247	251/127	97	124	30	48	65	14	7
Zhu, M. [26]	2009	Asian	China	PCR	Healthy	0.653	141/240	47	71	23	51	123	66	7
Pei, F. [20]	2009	Asian	China	PCR-SSP	Patients without CAD	0.854	234/216	82	107	45	42	108	66	8

Abbreviations: PCR, polymerase chain reaction; RFLP, restriction fragment length polymorphism; SSP, sequence-specific primer.

### Quantitative data synthesis

**Figure 2:**
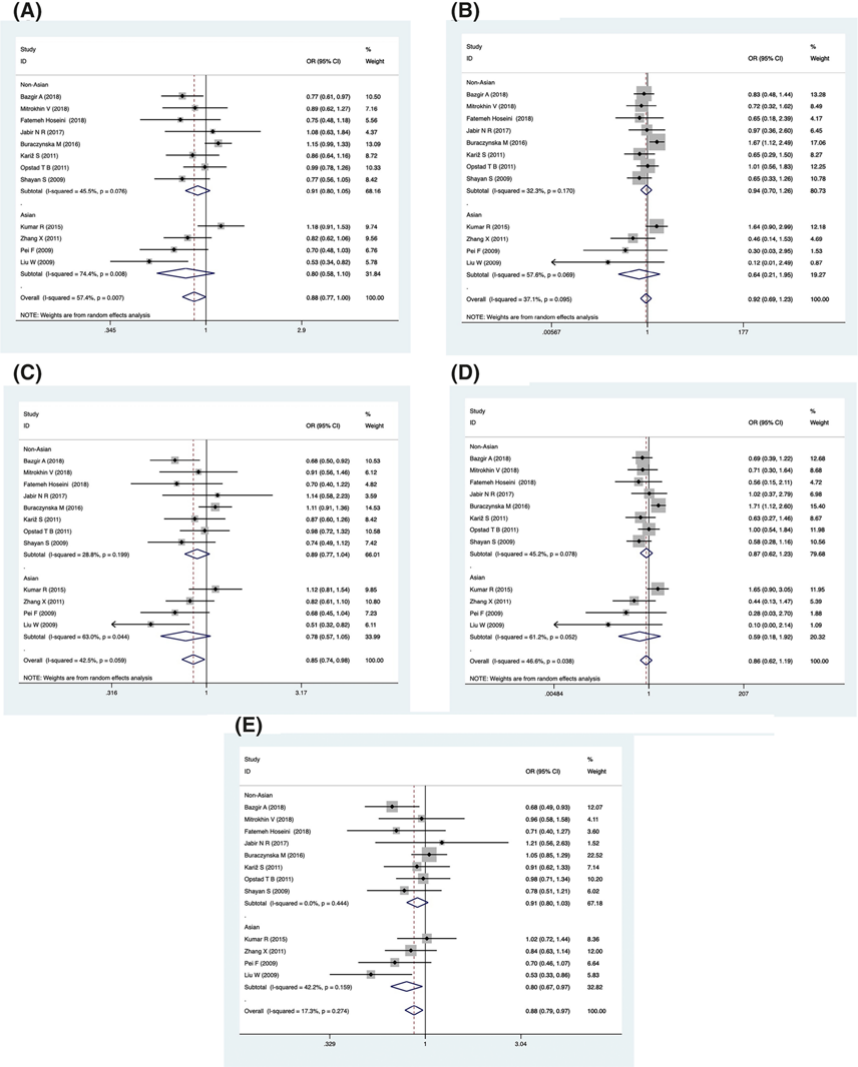
Forest plot describing the association between the -137 polymorphism (rs187238) and risk of across all study participants according to different genetic models (**A**) Allelic (C-allele vs. G-allele), (**B**) recessive (CC vs. GC + GG), (**C**) dominant (GC + CC vs. GG), (**D**) homozygous (CC vs. GG) and (**E**) heterozygous (GC vs. GG).

**Figure 3:**
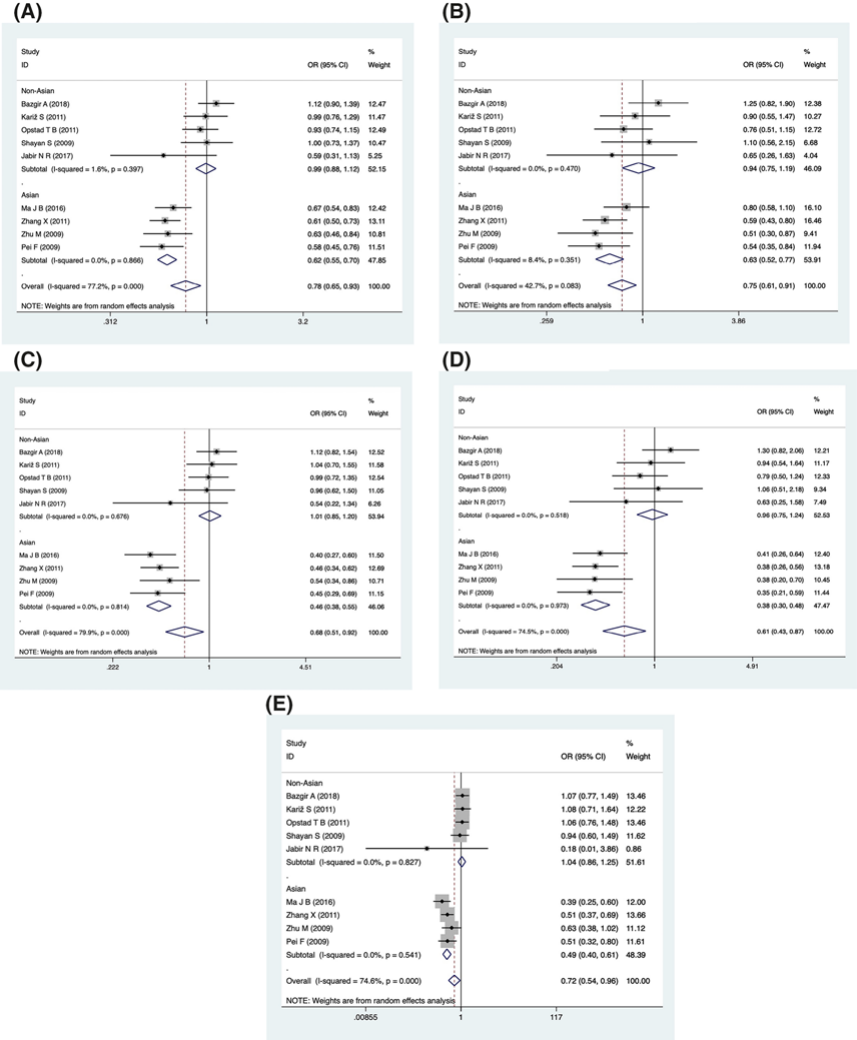
Forest plot describing the association between the -607 (rs 1946518) and risk of across all study participants according to different genetic models (**A**) Allelic (A-allele vs. C-allele), (**B**) recessive (AA vs. CA+ CC), (**C**) dominant (AA+CA vs. CC), (**D**) homozygous (AA vs. CC) and (**E**) heterozygous (CA vs. CC).

The results of the meta-analysis for the associations between the IL-18 promoter polymorphism -137 (rs187238), -607 (rs1946518) and CAD risks are shown in Table 3, and Figures 2 and 3. There were 12 eligible studies with 4450 cases and 3007 controls that focused on the association between the -137 polymorphism (rs187238) and CAD risk. Overall, significant associations were only revealed in the results under the genetic models of dominant and heterozygous. A decreased risk of CAD was observed in dominant and heterozygous genetic models. (GC+CC vs. GG, OR = 0.85, 95% CI = 0.74–0.98, *P*=0.024, *I^2^* = 42.5%; GC vs. GG, OR = 0.88, 95% CI = 0.79–0.97, *P*=0.012, *I^2^* = 17.3%) (Figure 2).

**Table 3:** Overall meta-analysis of the association between the -137 polymorphism (rs187238), IL-18-607 (rs1946518) and risk of CAD

Variables	*n*	C vs. G			CC vs. GC+GG			CC+GC vs. GG			CC vs. GG			GC vs. GG		
IL-18-137 (rs187238)		OR (95% CI)	*P*	*I^2^* (%)	OR (95% CI)	*P*	*I^2^* (%)	OR (95% CI)	*P*	*I^2^* (%)	OR (95% CI)	*P*	*I^2^* (%)	OR (95% CI)	*P*	*I^2^* (%)
Total	12	0.88 [0.77, 1.00]	0.050	57.4	0.92 [0.69, 1.23]	0.594	37.1	0.85 [0.74, 0.98]	0.024	42.5	0.86 [0.62, 1.19]	0.360	46.6	0.88 [0.79, 0.97]	0.012	17.3
**Ethnicity**																
Asian	4	0.80 [0.58, 1.10]	0.169	74.4	0.64 [0.21, 1.95]	0.433	57.6	0.78 [0.57, 1.05]	0.103	63.0	0.59 [0.18, 1.92]	0.382	61.2	0.80 [0.67, 0.97]	0.020	42.2
Non-Asian	8	0.91 [0.80, 1.05]	0.190	45.5	0.94 [0.70, 1.26]	0.670	32.3	0.89 [0.77, 1.04]	0.144	28.8	0.87 [0.62, 1.23]	0.427	45.2	0.91 [0.80, 1.03]	0.146	0
**HWE**																
HWE-Yes	10	0.84 [0.74. 0.95]	0.005	38.8	0.84 [0.62, 1.14]	0.272	16.4	0.81 [0.71, 0.92]	0.002	23	0.77 [0.57, 1.04]	0.089	20	0.82 [0.73, 0.92]	0.001	0
HWE-No	2	1.14 [0.99, 1.32]	0.074	0	1.54 [1.06,1.28]	0.022	0	1.11 [0.92, 1.35]	0.273	0	1.58 [1.08, 2.33]	0.020	0	1.06 [0.86, 1.29]	0.593	0

Variables	*n*	A vs. C			AA vs. CA+CC			AA+CA vs. CC			AA vs. CC			CA vs. CC		
IL-18-607 (rs1946518)		OR (95% CI)	*P*	*I^2^* (%)	OR (95% CI)	*P*	*I^2^* (%)	OR (95% CI)	*P*	*I^2^* (%)	OR (95% CI)	*P*	*I^2^* (%)	OR (95% CI)	*P*	*I^2^* (%)
Total	9	0.78 [0.65, 0.93]	0.006	77.2	0.75 [0.61, 0.91]	0.005	42.7	0.68 [0.51, 0.92]	0.011	79.9	0.61 [0.43, 0.87]	0.006	74.5	0.72 [0.54, 0.96]	0.025	74.6
**Ethnicity**																
Asian	4	0.62 [0.55, 0.70]	0.000	0	0.63 [0.52, 0.77]	0.000	8.4	0.46 [0.38, 0.55]	0.000	0	0.38 [0.30,0.48]	0.000	0	0.49 [0.40, 0.61]	0.000	0
Non-Asian	5	0.99 [0.88, 1.12]	0.872	1.6	0.94 [0.75, 1.19]	0.621	0	1.01 [0.85, 1.20]	0.903	0	0.96 [0.75, 1.24]	0.777	0	1.04 [0.86, 1.25]	0.627	0
**HWE**																
HWE-Yes	8	0.79 [0.65, 0.95]	0.014	79.6	0.75 [0.61, 0.93]	0.01	49.6	0.69 [0.51, 0.95]	0.021	82.3	0.61 [0.42, 0.89]	0.01	77.7	0.73 [0.54, 0.97]	0.032	77.2
HWE-No	1	0.59 [0.31, 1.13]	0.1111	/	0.65 [0.26, 1.63]	0.358	/	0.68 [0.51, 0.92]	0.185	/	0.63	[0.25, 1.58]	0.332	/	0.18 [0.01, 3.86]	0.274

However, in ethnicity subgroup analysis, significantly decreased CAD risks were only observed in Asian populations for heterozygous genetic models (GC vs. GG, OR = 0.80, 95% CI = 0.67–0.97, *P*=0.020, *I^2^* = 42.2%). When we restricted the analysis to HWE, a significant association with decreased CAD risk was not only observed in the dominant and heterozygous genetic models but also in the allele genetic model (GC+CC vs. GG, OR = 0.81, 95% CI = 0.71–0.92, *P*=0.002, *I^2^* = 23%; GC vs. GG, OR = 0.82, 95% CI = 0.73–0.92, *P*=0.001, *I^2^* = 0%, C vs. G, OR = 0.84, 95% CI = 0.74–0.95, *P*=0.005, *I^2^* = 38.8%).

For the -607 polymorphism (rs1946518), we included nine studies to analyze the association with CAD risk. These studies involving 2978 cases and 2309 controls were pooled into the meta-analysis. The overall OR with its 95% CI revealed a significantly reduced risk of CAD in all five genetic models (A vs. C, OR = 0.78, 95% CI = 0.65–0.93, *P*=0.006, *I^2^* = 77.2%; AA vs. CA+CC, OR = 0.75, 95% CI = 0.61–0.91, *P*=0.005, *I^2^* = 42.7%; AA+CA vs. CC, OR = 0.68, 95% CI = 0.51–0.92, *P*=0.011, *I^2^* = 79.9%; AA vs. CC, OR = 0.61, 95% CI = 0.43–0.87, *P*=0.006, *I^2^* = 74.5%, CA vs. CC, OR = 0.72, 95% CI = 0.54–0.96, *P*=0.025, *I^2^* = 74.6%) (Figure 3).

In subgroup analysis by ethnicity, reduced CAD risk was also found in five genetic models of the Asian population (A vs. C, OR = 0.62, 95% CI = 0.55–0.70, *P*=0.000, *I^2^* = 0%; AA vs. CA+CC, OR = 0.63, 95% CI = 0.52–0.77, *P*=0.000, *I^2^* = 8.4%; AA+CA vs. CC, OR = 0.46, 95% CI = 0.38–0.55, *P*=0.000, *I^2^* = 0%; AA vs. CC, OR = 0.38, 95% CI = 0.30–0.48, *P*=0.000, *I^2^* = 0%, CA vs. CC, OR = 0.49, 95% CI = 0.40–0.61, *P*=0.000, *I^2^* = 0%). When restricted to HWE, a significant association with decreased CAD risk was also observed in five genetic models (A vs. C, OR = 0.79, 95% CI = 0.65–0.95, *P*=0.014, *I^2^* = 79.6%; AA vs. CA+CC, OR = 0.75, 95% CI = 0.61–0.93, *P*=0.010, *I^2^* = 49.6%; AA+CA vs. CC, OR = 0.69, 95% CI = 0.51–0.95, *P*=0.021, *I^2^* = 82.3%; AA vs. CC, OR = 0.61, 95% CI = 0.42–0.89, *P*=0.010, *I^2^* = 77.7%, CA vs. CC, OR = 0.73, 95% CI = 0.54–0.97, *P*=0.032, *I^2^* = 77.2%).

**Figure 4:**
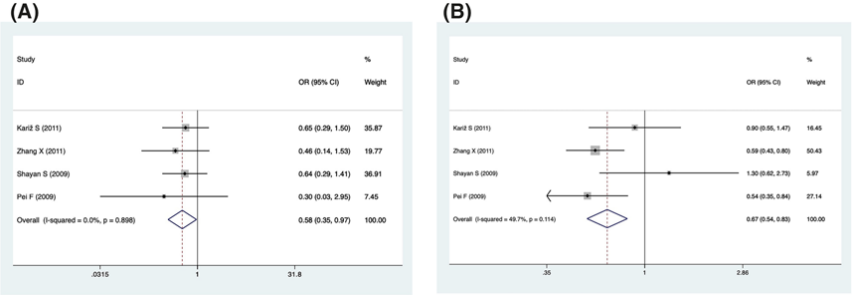
Forest plot describing the association between the IL-18 promoter polymorphism and risk of MI in recessive genetic model (**A**) For -137 polymorphism (rs187238); (**B**) for -607 polymorphism (rs 1946518).

CAD concludes many subtypes, such as myocardial infarction (MI), sudden death, angina and so on. MI causes more human deaths worldwide than any other disease. Therefore, we conclude the data that referred to the MI population and analyzed the relationship between MI risk factors and IL-18 promoter polymorphism (Tables 4 and 6, and  Figure 4). For different genetic models of the -137 polymorphism (rs187238), we observed significantly reduced risk in four genetic models: allelic, homozygous, dominant and recessive (C vs. G, OR = 0.81, 95% CI = 0.69–0.95, *P*=0.009, *I^2^* = 0%; CC vs. GC+GG, OR = 0.58, 95% CI = 0.35–0.97, *P*=0.036, *I^2^* = 0%; CC+GC vs. GG, OR = 0.80, 95% CI = 0.67–0.97, *P*=0.022, *I^2^* = 0%; CC vs. GG, OR = 0.55, 95% CI = 0.33–0.93, *P*=0.025, *I^2^* = 0%). However, the reduced risk of MI can only be observed in the recessive genetic model of the -607 polymorphism (rs1946518) (AA vs. CA+CC, OR = 0.67, 95% CI = 0.54–0.83, *P*=0.000, *I^2^* = 49.7%).

**Table 4:** Characteristics of studies which refer to MI

First author	Year	Ethnicity	Country	Genotyping method	Type of control	*P* for HWE	Cases/ Controls	Number of cases			Number of controls			Quality score
IL-18-137 (rs 187238)								GG	CG	CC	GG	CG	CC	
Kariž, S. [19]	2011	White	Slovenia	PCR- RFLP	Patients without CAD	0.439	169/326	90	71	8	162	141	23	8
Zhang, X. [27]	2011	Asian	China	PCR-SSP	Patients without CAD	0.301	468/432	352	112	4	308	116	8	6
Shayan, S. [25]	2009	Asian	Iran	PCR	Patients without CAD	0.768	136/140	64	61	11	60	63	17	7
Pei, F. [20]	2009	Asian	China	PCR-SSP	Patients without CAD	0.203	234/216	180	53	1	150	63	3	8

IL-18-607 (rs 1946518)								CC	CA	AA	CC	CA	AA	
Kariž, S. [19]	2011	Non-Asian	Slovenia	PCR- RFLP	Patients without CAD	0.895	169/326	55	86	28	109	158	59	8
Zhang, X. [27]	2011	Asian	China	PCR-SSP	Patients without CAD	0.616	468/432	170	210	88	90	220	122	6
Shayan, S. [25]	2009	Asian	Iran	PCR	Patients without CAD	0.247	130/127	53	59	18	48	65	14	7
Pei, F. [20]	2009	Asian	China	PCR-SSP	Patients without CAD	0.854	234/216	82	107	45	42	108	66	8

Abbreviations: PCR, polymerase chain reaction; RFLP, restriction fragment length polymorphism; SSP, sequence-specific primer.

**Figure 5:**
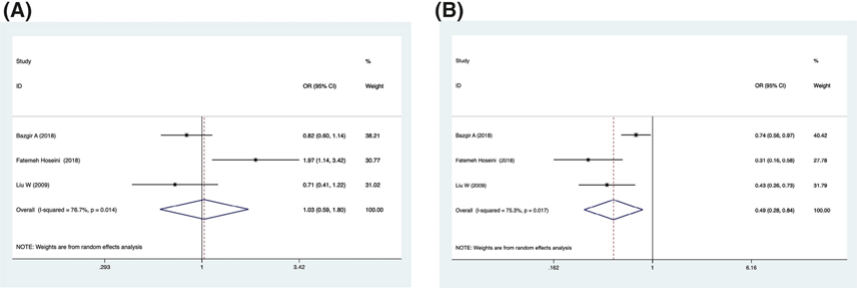
Forest plot describing the association between the -137 polymorphism (rs187238) and the number of stenotic coronaries in allelic genetic model (**A**) For SV group; (**B**) for MV group.

The number of stenotic coronary arteries is one of the indicators of the severity of coronary heart disease. We conclude data from three studies to analyze the correlation between the number of stenotic coronary artery and IL-18 promoter polymorphisms (Tables 5 and 7 and Figure 5). We defined only one coronary artery stenosis as a single-vessel group (SV), and two or more coronary stenosis was a multivessel group (MV). Table 7 shows that there was no significant correlation between the -137 polymorphism (rs187238) in the SV group, but in the MV group, we observed a reduced risk in the allelic, dominant and heterozygous models (C vs. G, OR = 0.49, 95% CI = 0.28–0.84, *P*=0.009, *I^2^* = 75.3%; CC+GC vs. GG, OR = 0.40, 95% CI = 0.23–0.70, *P*=0.001, *I^2^* = 68.7%; GC vs. GG, OR = 0.4, 95% CI = 0.24–0.68, *P*=0.001, *I^2^* = 62.9%).

**Table 5:** Characteristics of studies which refers to the number of stenotic coronaries

First author	Year	Ethnicity	Country	Genotyping method	Type of control	*P* for HWE	Cases/ Controls	Number of cases			Number of controls			Quality score
IL-18-137 (rs 187238)-SV								GG	CG	CC	GG	CG	CC	
Bazgir, A. [15]	2018	Asian	Iran	PCR-SSP	Healthy	0.183	198/364	112	68	18	160	171	33	7
Fatemeh, H. [23]	2018	Asian	Iran	PCR	Healthy	0.242	63/100	51	10	2	48	46	6	7
Liu, W. [21]	2009	Asian	China	PCR	Patients without CAD	0.236	152/145	128	24	0	99	44	2	6

IL-18-137 (rs 187238)-MV								CC	CA	AA	CC	CA	AA	
Bazgir, A. [15]	2018	Asian	Iran	PCR-SSP	Healthy	0.183	198/364	112	68	18	160	171	33	7
Fatemeh, H. [23]	2018	Asian	Iran	PCR	Healthy	0.242	63/100	51	10	2	48	46	6	7
Liu, W. [21]	2009	Asian	China	PCR	Patients without CAD	0.236	152/145	128	24	0	99	44	2	6

Abbreviations: PCR, polymerase chain reaction; RFLP, restriction fragment length polymorphism; SSP, sequence-specific primer.

**Table 6:** Overall meta-analysis of the association between the -137 polymorphism (rs187238), IL-18-607 (rs1946518) and risk of MI

Variables	*n*	C vs. G			CC vs. GC+GG			CC+GC vs. GG			CC vs. GG			GC vs. GG		
IL-18-137 (rs 187238)		OR (95% CI)	*P*	*I^2^* (%)	OR (95% CI)	*P*	*I^2^* (%)	OR (95% CI)	*P*	*I^2^* (%)	OR (95% CI)	*P*	*I^2^* (%)	OR (95% CI)	*P*	*I^2^* (%)
Total	4	0.81 [0.69, 0.95]	0.009	0	0.58 [0.35, 0.97]	0.036	0	0.80 [0.67, 0.97]	0.022	0	0.55 [0.33, 0.93]	0.025	0	0.84 [0.69, 1.01]	0.067	0

Variables	*n*	A vs. C			AA vs. CA+CC			AA+CA vs. CC			AA vs. CC			CA vs. CC		
IL-18-607 (rs 1946518)		OR (95% CI)	*P*	*I^2^* (%)	OR (95% CI)	*P*	*I^2^* (%)	OR (95% CI)	*P*	*I^2^* (%)	OR (95% CI)	*P*	*I^2^* (%)	OR (95% CI)	*P*	*I^2^* (%)
Total	4	0.75 [0.57, 1]	0.051	79.1	0.67 [0.54, 0.83]	0.000	49.7	0.65 [0.42, 1.01]	0.055	79.2	0.59 [0.33, 1.03]	0.062	76.9	0.68 [0.46, 1]	0.050	69.9

**Table 7:** Overall meta-analysis of the association between the -137 polymorphism (rs187238) and the number of stenotic coronaries

Variables	*n*	C vs. G			CC vs. GC+GG			CC+GC vs. GG			CC vs. GG			GC vs. GG		
IL-18-137 (rs 187238)		OR (95% CI)	*P*	*I^2^* (%)	OR (95% CI)	*P*	*I^2^* (%)	OR (95% CI)	*P*	*I^2^* (%)	OR (95% CI)	*P*	*I^2^* (%)	OR (95% CI)	*P*	*I^2^* (%)
SV	3	1.03 [0.59, 1.8]	0.920	76.7	0.58 [0.27, 1.24]	0.158	0	1.28 [0.53, 3.13]	0.584	83.7	0.64 [0.29, 1.38]	0.253	29.5	1.36 [0.56, 3.32]	0.503	83.3
MV	3	0.49 [0.28, 0.84]	0.009	25.3	0.85 [0.49, 1.47]	0.566	0	0.40 [0.23, 0.70]	0.001	68.7	0.64 [0.36, 1.12]	0.12	0	0.40 [0.24, 0.68]	0.001	62.9

### Sensitivity analysis

**Figure 6:**
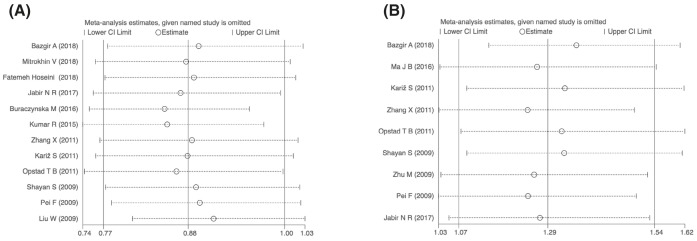
The influence of each study by removal of individual studies for allelic genetic model (**A**) For -137 polymorphism (rs187238); (**B**) for -607 polymorphism (rs 1946518).

For the -137 polymorphism (rs187238), the sensitivity analysis showed that no single individual study significantly affected the pooled OR in all genetic models. Additionally, for the -607 polymorphism (rs1946518), sensitivity analysis showed that none of the studies led to changes in the global ORs, indicating the robustness and stability of the results in this meta-analysis (Figure 6).

### Publication bias

**Figure 7:**
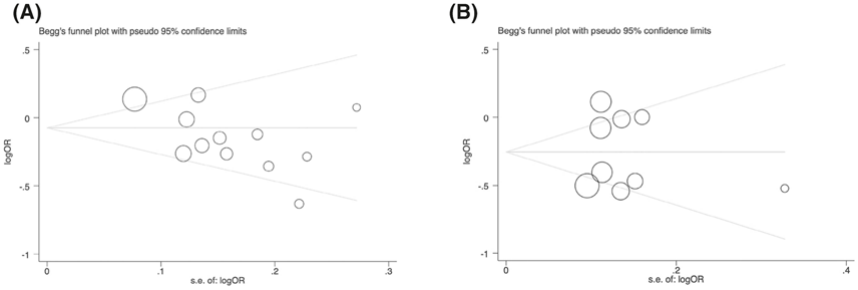
Begg’s funnel plot to assess publication bias in the meta-analysis of a potential association between IL-18 promoter polymorphism and risk of CAD in allelic genetic model (**A**) For -137 polymorphism (rs187238); (**B**) for -607 polymorphism (rs 1946518).

To evaluate the publication bias, Begg’s funnel plot and Egger’s test were performed. The *P*-values for Begg’s and Egger’s tests are shown in Table 8. Obvious publication bias was observed for the -137 polymorphism (rs187238) in allelic, homozygous and recessive models in Egger’s test. For the -607 polymorphism (rs1946518), there was no publication bias in all models. These results were also demonstrated by the shape of the funnel plot (Figure 7).

**Table 8:** Egger’s and Begg’s tests for the publication bias of IL-18-137 (rs 187238) and IL-18-607 (rs 1946518)

	Begg’s test *P*-value	Egger’s test *P*-value
IL-18-137 (rs 187238)		
C vs G	0.373	0.026
CC vs. GC+GG	0.064	0.004
CC+GC vs. GG	0.373	0.096
CC vs. GG	0.115	0.008
GC vs. GG	0.451	0.211
IL-18-607 (rs 1946518)		
A vs. C	0.917	0.972
AA vs. CA+CC	0.602	0.729
AA+CA vs. CC	0.917	0.599
AA vs. CC	0.348	0.563
AC vs. CC	0.466	0.513

## Discussion

IL-18 is a pleiotropic pro-inflammatory cytokine that affects both innate and acquired inflammatory responses [34,35], and it has been associated with the development of atherosclerosis by stimulating the production of atherogenic IFN-γ [36]. Previous studies have demonstrated that the concentration of IL-18 is higher in CAD patients, and the level of IL-18 could be an indicator to evaluate the risk of CAD [37,38]. A meta-analysis supported that circulating IL-18 was prospectively and independently associated with cardiovascular disease risk [39]. As the upstream of gene expression, the IL-18 promoter plays an important role in influencing the expression of IL-18. However, the relationship between IL-18 promoter polymorphisms and CAD is controversial.

In the present meta-analysis, for the -137 polymorphism (rs187238), we found that heterozygous and dominant models had a negative correlation with CAD. In addition, decreased CAD risks were only observed in Asian populations for heterozygous genetic models. This result is different from a previous meta-analysis by Dong et al. [40], which included only six studies. A recent study published by Mitrokhin et al. [28] did not find any relationship between the -137 polymorphism and CAD [28], and the same result was also reported by Kumar et al. [18], Kariž et al. [19], Pei et al. [20] etc. However, another study published an opposite conclusion: a significant increase in the G allele or GG genotype was observed in CAD patients. The present study also found that G-allele carriers in MV disease patients had a higher occurrence rate when compared with SV disease patients [21]. Thus, the -137 polymorphism (rs187238) not only correlated with CAD prevalence but also correlated with the severity of CAD. Therefore, we screened 12 studies and reanalyzed the relationship between the number of stenotic coronaries and the -137 polymorphism (rs187238). We also found that the -137 polymorphism (rs187238) correlated with MV disease. The allelic, heterozygous and dominant genetic models showed a reduced risk for MV disease. Another indicator to evaluate the severity of CAD is the type of CAD, MI is the most severe type of CAD, and we observed a significant correlation between MI and the -137 polymorphism (rs187238). Except for the heterozygous genetic model, the remaining four genetic models had a negative correlation with MI.

Previous studies have also shown that the genotype carrying the IL-18 promoter—607 C/A gene locus C was related to the high expression of IL-18, which leads to the up-regulation of cytokines, chemokines, adhesion molecules and matrix metalloproteinases [41,42]. Some studies have discussed the relationship between the -607 polymorphism (rs1946518) and CAD; however, the results are controversial. Shayan et al. [25] evaluated the role of two IL-18 gene polymorphisms at the -607(C/A) position in patients with CAD and healthy controls. They reported no significant association between genotypes and alleles and CAD. However, contrary conclusions were reported by other studies [17,10,26,27]. In our meta-analysis, we observed a significant association between the -607 polymorphism (rs1946518) and CAD, in which the -607 polymorphism (rs1946518) was negatively correlated with CAD. After subgroup analysis, this influence was only observed in the Asian population. It is worth noting that we observed a significant heterogeneity in overall analysis, but the heterogeneity was disappeared in ethnicity subgroup analysis. This results further indicate the -607 SNP is correlated with CAD risk for Asian population. Additionally, -607 SNP was also correlated with MI. However, unlike the -137 polymorphism (rs187238), only the recessive genetic model had a negative correlation with MI. Unfortunately, no study has revealed the -607 polymorphism (rs1946518) related to the number of stenotic coronaries.

To our knowledge, this is an update study focused on the association between IL-18 promoter polymorphisms and CAD risk, but it was the first meta-analysis evaluating the potential association of these two IL-18-related polymorphisms and the risk of MI and the number of stenotic coronaries. The strengths of our study are listed as follows: first, most of the genotype distributions in controls were consistent with ethnicity and HWE. Second, the relationship was analyzed using five types of genetic models, and the results were statistically significant. Third, the methodological issues for meta-analysis, such as Egger’s test, Begg’s funnel plots and subgroup analysis, were performed to ensure the stability of the results.

However, we also pay attention to the limitations in our meta-analysis. First, the small sample size of studies included was still inadequate, so the statistical power was reduced. Second, two studies did not conform to HWE expectations. Third, an obvious asymmetry in funnel plots and significant *P*-values for the -137 polymorphism (rs187238) through Egger’s test were found in the present study. In the present study, the small sample size may be an important reason for publication bias. Furthermore, studies only in English or Chinese have been searched. There might be studies in other languages that are not included, which might be another reason for the asymmetry. Last, in the study of the -137 polymorphism (rs187238), there are a few studies on Asian populations, and we expect more data on Asian populations.

## Conclusion

In conclusion, our results suggested that the -137 polymorphism (rs187238) and -607 polymorphism (rs1946518) of the IL-18 promoter were negatively associated with CAD, especially in the Asian population. In addition, some genetic models were correlated with the severity of CAD. However, the association between CAD and the -137 polymorphism (rs187238) should be interpreted with caution because of publication bias. Further detailed investigations involving larger, multiethnic samples are needed to clarify the role of these polymorphisms in CAD risk.

## Abbreviations

CAD: coronary artery disease
CHD: coronary heart disease
CI: confidence interval
HWE: Hardy–Weinberg equilibrium
IFN: interferon
IL-18: interleukin-18
MI: myocardial infarction
MV: multivessel
OR: odds ratio
SV: single-vessel
